# Protocol for a pilot randomised controlled trial of an intervention to increase the use of traffic light food labelling in UK shoppers (the FLICC trial)

**DOI:** 10.1186/s40814-015-0015-1

**Published:** 2015-06-06

**Authors:** Peter Scarborough, Charo Hodgkins, Monique M. Raats, Richard A. Harrington, Gill Cowburn, Moira Dean, Aiden Doherty, Charlie Foster, Edmund Juszczak, Anne Matthews, Anja Mizdrak, Cliona Ni Mhurchu, Richard Shepherd, Lada Tiomotijevic, Naomi Winstone, Mike Rayner

**Affiliations:** 1 Nuffield Department of Population Health, British Heart Foundation Centre on Population Approaches for NCD Prevention, University of Oxford, Oxford, UK; 2 Food, Consumer Behaviour and Health Research Centre, University of Surrey, Surrey, UK; 3 School of Biological Sciences, Queen’s University Belfast, Belfast, UK; 4 National Perinatal Epidemiology Unit, Nuffield Department of Population Health, University of Oxford, Oxford, UK; 5 National Institute for Health Innovation, University of Auckland, Auckland, New Zealand

**Keywords:** Food, Nutrition, Nutrition labelling, Traffic light labelling, Trial

## Abstract

**Background:**

Traffic light labelling of foods—a system that incorporates a colour-coded assessment of the level of total fat, saturated fat, sugar and salt on the front of packaged foods—has been recommended by the UK Government and is currently in use or being phased in by many UK manufacturers and retailers. This paper describes a protocol for a pilot randomised controlled trial of an intervention designed to increase the use of traffic light labelling during real-life food purchase decisions.

**Methods/design:**

The objectives of this two-arm randomised controlled pilot trial are to assess recruitment, retention and data completion rates, to generate potential effect size estimates to inform sample size calculations for the main trial and to assess the feasibility of conducting such a trial. Participants will be recruited by email from a loyalty card database of a UK supermarket chain. Eligible participants will be over 18 and regular shoppers who frequently purchase ready meals or pizzas. The intervention is informed by a review of previous interventions encouraging the use of nutrition labelling and the broader behaviour change literature. It is designed to impact on mechanisms affecting belief and behavioural intention formation as well as those associated with planning and goal setting and the adoption and maintenance of the behaviour of interest, namely traffic light label use during purchases of ready meals and pizzas. Data will be collected using electronic sales data via supermarket loyalty cards and web-based questionnaires and will be used to estimate the effect of the intervention on the nutrition profile of purchased ready meals and pizzas and the behavioural mechanisms associated with label use. Data collection will take place over 48 weeks. A process evaluation including semi-structured interviews and web analytics will be conducted to assess feasibility of a full trial.

**Discussion:**

The design of the pilot trial allows for efficient recruitment and data collection. The intervention could be generalised to a wider population if shown to be feasible in the main trial.

**Trial registration:**

ISRCTN: ISRCTN19316955

## Background

**Fig. 1:**
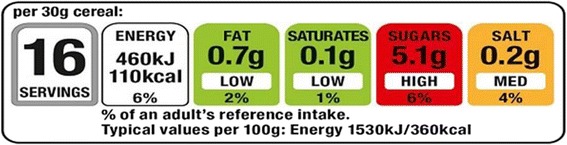
Example of front-of-pack (FOP) labelling that is being phased in by the participating supermarket chain. Source: participating supermarket website. Accessed October 2013

Front-of-pack (FOP) nutrition labelling has been used in various formats on foods sold in the UK since the mid-2000s, and the labels have a high penetration in the UK market [1]. FOP labels provide consumers with an ‘at a glance’ assessment of the nutritional quality of packaged foods. In October 2012, in an effort to unify FOP label formats, the UK Government announced its preferred system for FOP labelling, which has been accepted by many UK retailers and manufacturers [2]. This format incorporates traffic light labelling—a system that highlights the level of fat, saturated fat, total sugar and salt within a food with colour-coded indications of high (red), medium (amber) and low (green). The objective of traffic light labelling is to provide consumers with nutritional information and to make it easier for consumers to make healthier choices about the food they eat [3], and it represents an opportunity to intervene in order to influence purchasing behaviour. This paper describes a protocol for a pilot randomised controlled trial of an intervention aimed at increasing the use of traffic light labelling in order to encourage healthier purchasing decisions within food categories (specifically ready meals and pizzas). Figure 1 shows an example of traffic light labelling. Primary outcome data will be measured using electronic supermarket purchasing data in order to track changes in food purchasing behaviour. This pilot trial is being conducted as part of the Front of pack food Labelling: Impact on Consumer Choice (FLICC) study, supported by the National Prevention Research Initiative.

### Behaviour change theory in a food context

Within our initial desk research phase, identification of interventions previously performed which have addressed nutrition labelling as a component or outcome revealed that only a minority reported the use of a theoretical framework (manuscript under preparation). Of those reported, Social Cognitive Theory [4] was the most common with a range of other frameworks such as Theory of Planned Behaviour [5], Theory of Reasoned Action [6], Stages of Change Theory [7], Health Belief Model [8] and Theory of Meaningful Learning [9] being utilised to a lesser extent. While the importance of developing interventions based on a theoretical framework is widely accepted in behaviour change sciences, in contrast, it has been suggested that an atheoretical approach may be more appropriate in a food context [10–12] as no single theoretical framework appears to optimally fit the context in which food-related behaviour changes are required. However, by recognising that behaviour change frameworks tend to contain a limited range of overlapping mechanisms [13, 14], an approach involving selection of the most relevant mechanisms from the various frameworks has been suggested as preferable to approaching the design of an intervention from an atheoretical standpoint in a food context [15]. For this study, a theoretical approach based on selection of the relevant behaviour change mechanisms rather than the adoption of an entire theoretical framework was the approach that we chose to adopt.

### Rationale for food category focus

The focus of this trial is on purchases of chilled and frozen ready meals and pizzas, and the developed intervention is designed to change purchasing behaviour of these food categories. Ready meals have previously been defined as complete meals that require few or no extra ingredients, prepared by external procedures, and designed to replace the main course of a homemade main meal [16]. For the purposes of this trial, a ‘ready meal’ is defined as a pre-packaged chilled or frozen food item that consists of an individual pre-prepared meal or meal centre (excluding soups, breakfast cereals, quiches, sausage rolls, pasta pots, sandwiches and other deli counter items). Ready meals and pizzas have been chosen as the focus of the trial for a number of reasons; firstly, initial scoping research has demonstrated that these food items are highly likely to contain traffic light labelling. On an audit of a single supermarket in an affluent area with over 650 m^2^ of space in April 2013, 140 different ready meals and pizzas were observed, of which 115 (82 %) carried traffic light labelling. Additionally, government guidance recommends that FOP labelling should be provided on all ready meals [3]. Secondly, ready meals and pizzas have considerable nutritional variance. Table 1 shows the distribution of traffic light colours for the 373 ready meals and pizzas in the participating supermarkets’ own brand as of October 2013—the audit revealed that across the categories, green, amber and red lights were all available for each of the four nutrients included in the traffic light labels. Similarly, an analysis of 300 chilled and frozen ready meals from 20 different manufacturers showed considerable variation in fat, saturates, sugar and salt levels [17]. Thirdly, ready meals contribute a large and growing proportion of food sales in the UK. The total market of ready meals in the UK was estimated to be worth £2.8 billion for over 500 million kg of food in 2012, with increases in both metrics expected by 2017 [18].

**Table 1:** Distribution of traffic light colours on 373 own-brand ready meals and pizzas from the participating supermarket

	Number (%) of foods with red, amber or green lights			
	Total fat	Saturated fat	Total sugar	Salt
Red	88 (23.4)	173 (46.4)	24 (6.4)	50 (13.4)
Amber	198 (53.1)	81 (21.7)	28 (7.5)	278 (74.5)
Green	87 (23.3)	119 (31.9)	321 (86.1)	45 (12.1)

### Study objectives

The goal of this pilot RCT is to assess the feasibility of a full RCT to measure the effectiveness of an intervention designed to help people use traffic light food labels to purchase healthier ready meals and pizzas. To achieve this goal, the pilot RCT is designed to meet the following objectives:

1. To obtain reliable estimates regarding recruitment, retention and data completion.
2. To produce estimates of the potential effect size (mean and standard deviation (SD)) of the web-based intervention on purchases of ready meals and pizzas (primary outcome).
3. To produce estimates of the potential effect size (mean and SD) of the intervention on purchases of all foods, purchases of fruits and vegetables, and psychosocial variables associated with label use (secondary outcomes).
4. To conduct a process evaluation consisting of semi-structured interviews and web analytics to explore the acceptability of the trial to both participants and the participating supermarket chain, to explore unintended consequences of the intervention and to explore the take up of different elements of the intervention.

The underlying hypotheses that will be tested in the full trial are that an intervention designed to help people use traffic light labels to buy healthier ready meals and pizzas will

- H1: increase the healthiness of purchased ready meals and pizzas;
- H2: not affect the total number (on average) of ready meals and pizzas typically purchased;
- H3: not change purchasing behaviour outside of the targeted food category (ready meals and pizzas); and
- H4: operate by impacting on mechanisms affecting belief and behavioural intention formation as well as those associated with planning and goal setting and the adoption and maintenance of the behaviour of interest, namely traffic light label use during purchases of ready meals and pizzas.

The intervention has been designed to be food category-specific, focussing only on ready meals and pizzas and therefore we hypothesise that it will only impact on food choices within this category. This is the most likely outcome since many of the other food categories do not have sufficient coverage of front-of-pack nutrition labelling for the consumer to use in the way described by the intervention. However, the pilot nature of this study means that it will be possible to explore whether the effect ‘spills over’ to other food categories utilising the secondary outcome variables.

The study objectives closely follow the ‘Assessing feasibility and piloting methods’ section of the Medical Research Council’s guide to developing and evaluating complex interventions [19].

## Methods/design

**Fig. 2:**
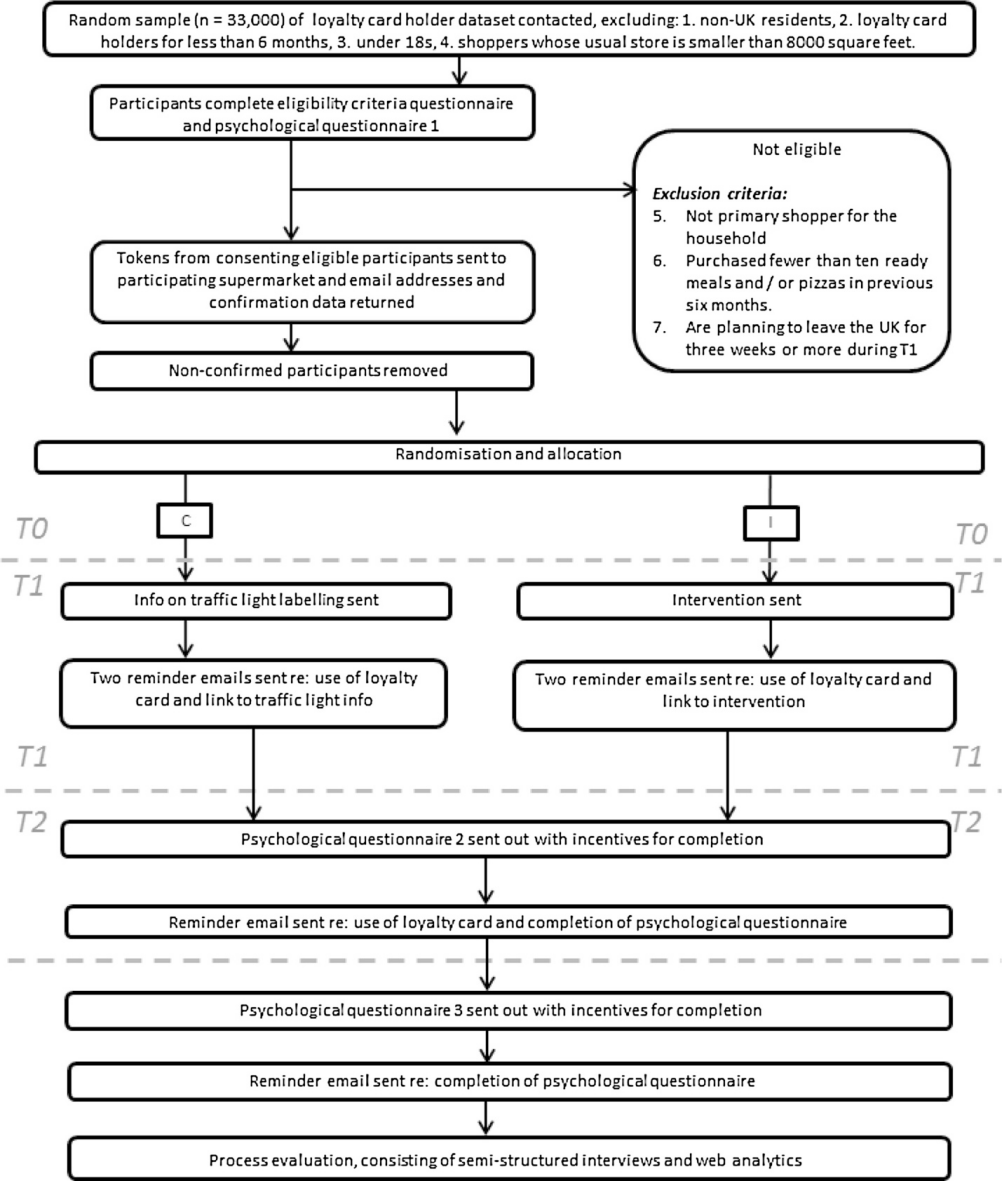
Flow chart of study design

The design is a two-arm parallel randomised controlled trial comparing the intervention against information about traffic light labelling. Data collection will take place over 44 weeks, with 26 weeks of baseline data (−T1), 6 weeks of intervention (T1) and 12 weeks of follow-up without intervention (T2), with questionnaire data collected at recruitment (T0) and during the intervention (T1) and follow-up (T2) periods. Figure 2 shows how the trial will progress, and Table 2 gives a timetable for data collection.

**Table 2:** Timetable of data collection

	−T1	T0	T1	T2	Post-study
	Baseline data	Recruitment	Intervention	Wash out	
	(26 weeks)	(4 weeks)	(6 weeks)	(12 weeks)	
Data collection					
Electronic sales data	X		X	X	
Psychosocial questionnaires		X	X	X	
Process evaluation: semi-structured interviews					X
Process evaluation: web analytics		X	X	X	

### Setting

Participants will be drawn from the participating supermarket loyalty card database, which covers all regions of the UK. Data collection will take place in any participating supermarket in the UK when a participant makes a food purchase using their loyalty card, wherever participants access the study questionnaires, through phone interviews as part of a process evaluation and through web analytics as part of an assessment of interaction with the intervention.

### Participants

Participants will be recruited from the database of loyalty card holders held by the participating supermarket chain. This database contains approximately 1.1 million people. Where a loyalty card is shared by multiple users, only one user will be contacted for recruitment. The exclusion criteria are shown in Fig. 2. The recruitment email will only be sent to loyalty card holders who meet inclusion criteria 1, 2, 3 and 4. Eligibility for the three remaining criteria will be assessed using responses to the screening questionnaire. Inclusion criterion 4 requires participants to regularly shop at large supermarkets—this is to ensure that they have access to a wide range of ready meals and pizzas.

### Recruitment and allocation strategy

A recruitment email will be sent to randomly selected loyalty card holders by the participating supermarket. The email will include eligibility criteria, brief details of the study and a link to the study website, where participants can access details about the study, complete an eligibility screening questionnaire, provide consent to be included in the study and complete the baseline demographics questionnaire. Participants will be asked to provide their loyalty card membership number. Participants will then be directed to the first of the psychosocial questionnaires which will measure variables related to traffic light labelling use. Responses will be sent to the research team, who will submit the membership numbers of enrolled participants to the supermarket chain. All enrolled participants will then be randomly allocated to intervention or control. A similar recruitment method with the same participating supermarket and the same study population has been used for a previous web-based study of how people use traffic light labelling of foods to make healthier choices (manuscript under preparation). This experience has provided the FLICC study team with an estimate of likely recruitment rates for the pilot RCT.

Block randomisation will be used stratified by gender and whether or not participants have dependent children to allocate individuals to the intervention or control arm. The randomisation process will be restricted to only two of the study team (EJ and RH), one of whom is the director of the clinical trials unit for the National Perinatal Epidemiology Unit. Researchers will be blind to the randomisation process. Participants in both arms of the trial will be aware that the study is about healthy food purchasing and traffic light food labelling. All participants will be sent a URL to a password-protected web application, which will remain open to the participants for 6 weeks (during T1). Participants in the intervention arm will have access to the intervention via this URL (described below). Participants in the control arm will be provided access (via the URL) to only one element of the intervention—a description of traffic light labels on foods.

All participants will be asked to complete two further psychosocial questionnaires similar to that used at T0. A link to the questionnaires will be emailed at the end of the intervention period (T1) and directly after completion of the follow-up period (T2). Participants will receive a £10 electronic gift voucher (that cannot be used in the participating supermarket) for completing the questionnaire at T1 and a further £10 electronic gift voucher for completing the questionnaire at T2. Four reminder emails will be sent to all participants throughout the course of the trial. The first two reminder emails will be sent during T1 (in weeks 3 and 5) reminding all participants to use their loyalty card for food purchases, directing the control arm to information about traffic light labels and directing the intervention arm to the intervention. The remaining two emails will be sent a week after the follow-up psychological questionnaires are sent out, in order to remind participants to complete them.

Measuring recruitment, retention and data completion rates is an objective of the study—for the purpose of recruiting for the pilot trial, we are assuming the following:

- Recruitment rate of 5 %—based on recruitment for a previous study using the same method and population which had a recruitment rate of 3.8 % (manuscript under preparation), adjusted for web-based recruitment with incentives [20].
- Loss of 20 % of consenting participants due to failure to meet eligibility criteria 5, 6 or 7 (estimate to be refined from pilot data).
- Retention rate of 93 %—based on retention rates for the SHOP trial, which collected 6 months of electronic supermarket sales data [21].
- Questionnaire data completion rate of 30 %—based on a 25 % completion rate for follow-up of web-based questionnaires [22] adjusted for web-based retention with incentives [20].

We are aiming to receive approximately 400 complete sets of psychosocial questionnaire data and 1200 complete sets of electronic sales data. This will allow us to detect an effect size (measured by Cohen’s d statistic) of 0.28 for the psychosocial questionnaire data and 0.16 for the electronic sales data. In general, effect sizes of 0.2 or less are considered ‘small’. The data collected in the pilot trial will allow us to refine our sample size estimates for the main trial. According to the assumed rates detailed above, we will recruit approximately 1300 participants, which will require us to email 33,000 loyalty card holders, from a total database of over 1 million. Should recruitment rates be lower than expected, further recruitment emails will be sent out.

Each of these assumed rates will be tested in the pilot trial, and the recruitment strategy may not result in the estimated number of participants. For example, it is not clear whether UK supermarket shoppers consistently use loyalty cards as much as in New Zealand, where the estimate of retention rate originates.

### Consent

All participants will complete an online consent form before being included in the study, which will be accessible from the website link in the recruitment email. All potential participants will have to review a participant information sheet before enrolling, indicating the nature of the study and the implications of participation. Participants will be informed that they may withdraw or unsubscribe from the study at any point without giving a reason. Unsubscribed participants will receive no further contact from the study team, but food purchase data will continue to be collected. Those participants who withdraw will receive no further contact and electronic sales data will be censored at the withdrawal date. Participants will express their desire to withdraw or unsubscribe by calling a UK landline during office hours or by contacting a dedicated email address.

### Intervention design

To improve people’s food choices, previous interventions have generally fallen into two main groups: (a) interventions that encourage healthier eating at a diet level, i.e. eat more fruits and vegetables and less fatty foods etc. (*inter-category*); (b) interventions that encourage use of nutritional labels to improve choice between similar products (*intra-category*). In terms of how people make decisions in real world environments, we know that they typically use simple heuristics to minimise the amount of information they process [23]; in a food context, this may equate to a simple rule of thumb such as ‘an apple is healthier than a chocolate bar’. This is a decision making strategy that fits relatively well with the types of interventions in the first group above which focuses on inter-category shifts in food purchase; however, this strategy does not help when comparing between two foods in the same category which are described similarly, e.g., ready meal A and ready meal B. In order to make these more difficult intra-category decisions, interventions have tended to instruct people to compare the risk nutrient content of products. This involves switching to a systematic processing approach from a simple heuristic approach [23], and in a shopping environment, people tend not to have the resources (i.e. time, effort, motivation) to use a systematic processing approach. Furthermore, the number of cues they have to process to reach a decision increases, and they are often presented with conflicting cues across the risk nutrients (e.g. ready meal A—fat is high but salt is low vs ready meal B—fat is low but salt is high). Therefore, what people are being asked to do requires significant investment of time, effort and motivation and is a clear departure from their typical choice behaviour.

This intervention aims to help people make *intra-category* decisions (i.e. to compare ready meal A and ready meal B) and by focussing only on the use of the traffic light element of the nutrition label, aims to reduce the amount of systematic processing required. By acknowledging the complexity involved with changing food-related behaviour and recognising the need for multiple mechanisms to support behaviour change in a food domain, the proposed intervention will impact on mechanisms affecting belief and behavioural intention formation and as well as those associated with planning and goal setting and the adoption and maintenance of the behaviour of interest, namely traffic light label use during purchases of ready meals and pizzas.

Mechanisms of behaviour change can be defined as being the means by which the techniques employed by an intervention are expected to impact on behaviour. For example, by providing information on the consequences of a particular behaviour to an individual in an intervention, one might expect to achieve the desired behavioural change via the mechanism of ‘belief formation’. Therefore, by selecting the most relevant behaviour change techniques [24] and aligning those with the previously identified mechanisms that might best achieve the desired outcomes of this study, the components included in the final FLICC intervention were developed.

The resultant intervention, delivered by a web application, will take the participant through a series of sections, which are designed to impact on the identified mechanisms of behaviour change. These sections are detailed in Table 3. Some of the sections of the web application are passive, where the web application is used as a tool to deliver information to the participant. Some of the sections are interactive, where the participant is encouraged to engage with the web application. The passive and interactive elements of the intervention are highlighted in Table 3.

**Table 3:** Intervention components

Behaviour change techniques	Behavioural mechanisms impacted	Intervention components
Provide information on consequences of behaviour to the individual	Mechanisms affecting belief formation/Cognitive mechanisms	The risks of eating a diet high in fat, saturated fat, salt sugar and the prominence of these nutrients in ready meals are pizzas are reported (passive)^a^.
	● Attentions bias	Personalised feedback on the traffic light profile of the 6 months of ready meals and pizzas purchased by the participant in − T1 study period are delivered. Participants will be presented with an infographic summarising the 6 months of data and will be able to interrogate the previous data in simple tables, with comparisons made to other available products (interactive).
	● Optimistic bias	
Provide instruction (how to perform the behaviour)	Mechanisms of intention formation	Information about the traffic light label profile of a selection of the ready meals and pizzas that are available from the participating supermarket will be provided in tabular form that the participant can interrogate, designed to highlight the potential for nutritional improvement within the ready meals and pizzas categories (interactive).
	● Outcome expectancies	
	● (Action) self-efficacy	
	● Perceived behavioural control	
	● Heuristics	
Goal setting	Planning and goal setting	The following outcome goal is provided: ‘Use traffic light labels when you are shopping in (participating supermarket) for ready meals and pizzas. Compare the traffic light labels between products and try to buy healthier ready meals and pizzas than you would normally. You can do this by: reducing the number of red lights on the label and increasing the number of green lights on the label’ (passive).
Modelling the behaviour	Mechanisms of intention formation	A short video showing individuals performing the behaviour in a real store will be provided (passive).
	● Outcome expectancies	
	● (Action) self-efficacy	
	● Perceived behavioural control	
Prompt practice	Mechanisms of intention formation	An experiential task will be provided which allows participants to increase their self-efficacy in using traffic light food labels. This will consist of multiple choice tests asking participants to choose healthier versions of ready meals or pizzas with and without traffic light information provided. The intention is to demonstrate that the traffic light information can make these decisions easier to make (interactive).
	● (Action) self-efficacy	
	● Perceived behavioural control	
Action planning	Planning and goal setting	Participants will be encouraged to plan when and where they will perform the desired behaviour via the development of intention statement(s) which will be entered into the web application by the participant (interactive).
Provide feedback on performance	Adopting and maintaining behaviour	Participant is provided with data on performance against the desired behavioural goal at the end of the trial period. This will be in the form of the infographic used in the ‘personalised feedback’ section, and will be provided with a comparison against the 6 months of shopping conducted in T -1. Participants will be informed in T1 that this feedback will arrive (passive).

^a^This element will be provided to participants in both the intervention and the control arm

### Outcome measures

#### Recruitment, retention and data completeness

Participants will be deemed to have been fully retained in the study if (a) they do not contact the study team to withdraw or unsubscribe and (b) if psychosocial questionnaires sent at T1 and T2 are completed. Partial retention rates for those who only complete the questionnaire at T1 or who only contribute food purchase data will also be calculated. Recruitment and retention rates from different socioeconomic groups (measured by area-level deprivation) will be assessed by comparison of the socioeconomic profile of the recruited sample with the profile of the whole loyalty card database from which the sample was drawn.

#### Effect sizes

The research team will receive electronic sales data for all food purchases during the study period from the participating supermarket at two stages: after allocation and after completion of the study. The primary outcome measures for the main trial will be healthiness of ready meals and pizzas that carry traffic light labelling. For each participant, mean healthiness of all ready meals and pizzas purchased during the entire time period will be assessed in T-1, T1 and T2 and differences between intervention and control arms at T1 and T2 will be controlled for differences at baseline (T-1). Purchase data will be collected via electronic sales data linked to participants’ loyalty cards. Comparisons between intervention and control at T1 will measure the immediate effect of the intervention, and at T2 will measure whether the effect is sustained for the following 12 weeks after the intervention is removed. The ‘healthiness’ of each purchased ready meal or pizza will be a combination of the information provided on the traffic light label, weighted by factors derived from a parallel choice experiment assessing the importance of different elements of the label (manuscript under preparation). Details of how the scale is constructed are provided in the appendix.

Secondary outcome measures (assessed as difference in means and SD between intervention and control) will be

1. Number of ready meals and pizzas purchased in T2/T1.
2. Amount (£) of ready meals and pizzas purchased in T2/T1.
3. Total amount (g) of fat, saturated fat, sugar and salt in ready meals purchased in T2/T1.
4. Amount (£) of all foods purchased in T2/T1.
5. Amount (£) of fruit and vegetables purchased in T2/T1.
6. Psychosocial variables including beliefs, attitudes, intention, outcome expectancies and procedural knowledge measured in T2/T1.

Secondary outcome measure 3, in combination with the primary outcome measures, will allow us to calculate clinically meaningful minimum differences for a sample size calculation for a full trial.

The data collected from the loyalty cards and the questionnaires will be stored in an anonymised dataset held on servers owned and maintained by the study sponsor for a period of 15 years, after which all datasets will be deleted.

#### Process evaluation: semi-structured interviews

A process evaluation will be conducted after completion of the data collection. Qualitative data will be collected from approximately five telephone interviews with representatives of the participating supermarket and ten interviews with participants from both arms. Participants will be asked whether they are willing to be part of the process evaluation at recruitment, and those that take part will be provided with a £10 voucher. Recruitment of the supermarket staff to the telephone interviews will be via direct contact with key staff with input to the project. The telephone interviews will be semi-structured and will probe for information about the mechanism of delivery of the intervention, data collection, data transfer, acceptability of the interventions, and feasibility of rolling out the study design to a full trial. The interviews will be digitally recorded and transcribed.

#### Process evaluation: web analytics

The use of web analytics to track how people interact with web-based health interventions has been demonstrated to be a worthwhile contribution to process evaluations, providing quantitative data alongside the qualitative data collected by the semi-structured interviews [25]. We will use a combination of Piwik analytics (http://piwik.org/) or a similar package, and custom built analytics tools, which will allow us to link visits to the intervention website to unique participant id codes. The analytics package will be used to measure the following variables for each participant:

- The number of visits to the intervention website,
- The number of visits to each webpage within the intervention,
- The average length of time spent visiting the website,
- Completion of the intervention (i.e. whether all of the sections of the intervention are visited across all visits),
- Responses to the experiential task (i.e. whether the participant gets the correct or incorrect answer in the experiential task),
- The internet browser and operating system used by the participant (this will allow us to identify the proportion of participants visiting the intervention on a smart phone or handheld device),
- Pathway used by participant (i.e. how they navigated the website),
- The time and date of each visit.

These data will be used to analyse how the participants engaged with the different elements of the intervention and to identify potential areas of improvement, both in terms of content and structure.

### Statistical analysis

Demographic characteristics and outcomes data will be summarised with counts and percentages for categorical variables, means (standard deviations) for normally distributed continuous variables and medians (with interquartile or simple ranges) for other continuous variables. At time points T1 and T2, repeated measures ANCOVA will be used to assess differences between intervention and control arms, adjusted for gender, dependent children and baseline measures [26]. If outcome data are not normally distributed, then differences will be assessed either using transformed data or by using appropriate non-parametric tests. Results will be presented as point estimates accompanied by 95 % confidence intervals. Analyses will be conducted on an ‘intention to treat’ basis (i.e. data for participants who unsubscribe from the study will be used in the final analyses). Subgroup analyses by socioeconomic status will be conducted to assess potential impact of the intervention on social inequalities. Since this is a pilot study with a sample size based on a small effect size and unclear recruitment rates, it is not guaranteed that the study will be adequately powered to detect differences between intervention and control arms, particularly in sub-analyses. The socioeconomic status of the participants will be compared with that of the loyalty card database from which they are drawn to assess inequalities in recruitment, using area-level deprivation measures.

## Discussion

The FLICC pilot trial is the first example of an experimental study to increase the use of traffic light labels via a behaviour change intervention in a real world supermarket setting. The randomised experimental design of this study is essential in order to isolate the effect of the designed intervention in a supermarket setting, where there are many competing factors that affect purchasing decisions (e.g. price promotions, product placement, seasonal food availability). The use of electronic sales data for measuring changes in food purchasing behaviour has been used in previous supermarket-based trials [21, 27–29]. The advantage of this data collection method is that it passively measures food purchase behaviour, i.e. is not based on self-report or reliant on actions of the participant. The electronic nature of the data collection reduces the burden on both the participants and the study co-ordinators. Limitations of supermarket loyalty card data include that loyalty cards can be shared by multiple users, and it is impossible to link purchases with specific users, and loyalty cards may not be used for all purchases during the data collection period. Additionally, not all supermarket purchasing may be conducted within the same chain of supermarkets. In the FLICC trial, participants will be encouraged and reminded to use their loyalty cards throughout the study and we will only recruit individuals who describe themselves as the primary shopper for a household, and we will investigate these limitations further in the process evaluation. Another limitation is that supermarket purchases are not a direct measure of food consumption; however, supermarket till receipts have previously been shown to correlate well with energy and fat consumption levels collected by food diaries [30–32].

The data collection, recruitment strategy and the intervention delivery for the FLICC trial are all based on remote methods which do not require face-to-face contact between the participants and the study co-ordinators. This removes any requirement for the trial to be geographically based in a single area, which increases the size of our potential population. The automated delivery of the intervention and collection of data reduces the cost of the study, and if the intervention is shown to be effective in a full trial, then it has the potential to be easily rolled out to a wider population by any supermarket that collects data using loyalty cards.

After the recent government recommendation for FOP food labelling, traffic light labelling of foods is on the increase in the UK with major retailers and manufacturers pledging to introduce the labels in 2014 [2]. Interventions aimed at helping people to use these tools to improve the healthiness of their shopping are needed in order to fully utilise their potential, and policy makers have a need to measure the impact that the provision of FOP food labelling can have on food purchase decisions in a real world setting now that these labels are becoming more widely available on food packaging.

## Trial status

The pilot randomised controlled trial is registered at the International Standard Randomised Controlled Trial Register, with id number ISRCTN19316955. The project has received ethical approval from the Central University Research Ethics Committee of the University of Oxford (reference number: SSD/CUREC1/14-008) and the University of Surrey Ethics Committee (reference number: EC/2014/153/FAHS). We are aiming to recruit for the pilot trial in Spring 2015. Results will be disseminated in articles submitted to peer-reviewed journals in late 2015.

### Sponsorship and funding

The pilot trial is supported by a research grant from phase IV of the National Prevention Research Initiative (MR/J000256/1) and is sponsored by the University of Oxford (Clinical Trials and Research Governance, Joint Research Office, Block 60, Churchill Hospital, Old Road, Headington, Oxford, OX3 7LE).

## Figures and Tables

**Fig. 3:**
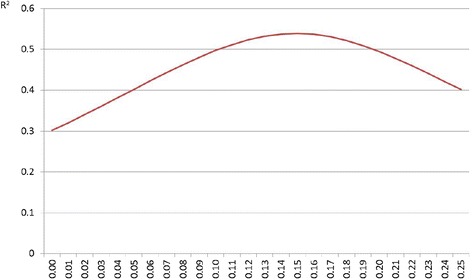
The fit (*R*^2^) of logistic regression models varying the amber score for the healthiness scale

## Appendix

### Derivation of the ‘healthiness’ scale

Formally, the healthiness scale will be constructed as follows:

$$ {H}_x=0.25{N}_G+0.15{N}_A $$

Where

- *H*_*x*_ is the healthiness score for food *x*
- *N*_*G*_ is the number of green traffic lights for food *x*
- *N*_*A*_ is the number of amber traffic lights for food *x*

The healthiness scale is designed so that the least healthy traffic light (four red lights) scores zero and the healthiest traffic light (four green lights) scores one. Hence, the constant applied to the number of green lights is 0.25 and the constant applied to the number of red lights is zero. The relative weighting for amber traffic lights (0.15) was derived from a choice experiment, where a sample of 183 UK shoppers were asked to select which they think is the healthiest traffic light label from a series of 20 pairwise comparisons. Figure 3 shows the fit of a logistic regression where the odds of choosing a label is estimated on the basis of the difference in healthiness between the two labels, where healthiness is defined using the equation above, but the constant applied to the number of amber lights is allowed to vary between zero and 0.25. The best fit to the data occurs when the constant is 0.15, indicating that the sample of shoppers considered amber lights to be closer to green lights than to red lights.
